# Effects of air pollution control policies on intracerebral hemorrhage mortality among residents in Tianjin, China

**DOI:** 10.1186/s12889-023-15735-3

**Published:** 2023-05-11

**Authors:** Jiahui Xu, Xiaolin Yin, Tingting Jiang, Shiyu Wang, Dezheng Wang

**Affiliations:** 1 School of Public Health, Tianjin Medical University, Tianjin, China; 2 NCDs Preventive Department, Tianjin Centers for Disease Control and Prevention, No. 6 Huayue Road, Hedong District, Tianjin, 300011 China

**Keywords:** Intracerebral hemorrhage, Air pollution, Policy evaluation, Interrupted time series analysis, Autoregressive integrated moving average models

## Abstract

**Background:**

Exposure to air pollution is an important risk factor for intracerebral hemorrhage (ICH), which is a major cause of death worldwide. However, the relationship between ICH mortality and air quality improvement has been poorly studied. This study aims to evaluate the impact of the air pollution control policies in the Beijing-Tianjin-Hebei region on ICH mortality among Tianjin residents.

**Methods:**

This study used an interrupted time series analysis. We fitted autoregressive integrated moving average (ARIMA) models to assess the changes in ICH deaths before and after the interventions of air pollution control policies based on the data of ICH deaths in Tianjin collected by the Tianjin Center for Disease Control and Prevention.

**Results:**

Between 2009 and 2020, there were 63,944 ICH deaths in Tianjin, and there was an overall decreasing trend in ICH mortality. The intervention conducted in June 2014 resulted in a statistically significant (*p* = 0.03) long-term trend change, reducing the number of deaths from ICH by 0.69 (95% confidence interval [CI]: -1.30 to -0.07) per month. The intervention in October 2017 resulted in a statistically significant (*p* = 0.04) immediate decrease of 25.74 (95% CI: -50.62 to -0.85) deaths from ICH in that month. The intervention in December 2017 caused a statistically significant (*p* = 0.04) immediate reduction of 26.58 (95% CI: -52.02 to -1.14) deaths from ICH in that month. The intervention in March 2018 resulted in a statistically significant (*p* = 0.02) immediate decrease of 30.40 (95% CI: -56.41 to -4.40) deaths from ICH in that month. No significant differences were observed in the changes of male ICH mortality after any of the four interventions. However, female ICH deaths showed statistically significant long-term trend change after the intervention in June 2014 and immediate changes after the interventions in December 2017 and March 2018. Overall, the interventions prevented an estimated 5984.76 deaths due to ICH.

**Conclusion:**

During the study period, some interventions of air pollution control policies were significantly associated with the reductions in the number of deaths from ICH among residents in Tianjin. ICH survivors and females were more sensitive to the protective effects of the interventions. Interventions for air pollution control can achieve public health gains in cities with high levels of air pollution.

## Background

Stroke is the second leading cause of death globally, resulting in over 6 million deaths annually, including 2 million in China [1]. Intracerebral hemorrhage (ICH) is the second most common subtype of stroke, characterized by high morbidity, disability, and mortality. It causes a severe burden on patients and society in China and worldwide [2, 3].

In China, hemorrhagic stroke comprises a greater proportion of all stroke cases compared to in western countries [1]. In 2016, the incidence of ischemic stroke in China was approximately twice that of hemorrhagic stroke, but hemorrhagic stroke had a higher mortality rate than ischemic stroke [4]. Moreover, hemorrhagic stroke contributed more to years of life lost (YLL) and disability adjusted life years (DALYs) than ischemic stroke, indicating that hemorrhagic stroke poses a greater risk of premature death for the Chinese population [5].

The 2015 Global Burden of Disease Study analyzed the 25 years of data and found that air pollution significantly affects DALYs and mortality caused by ischemic heart disease, cerebrovascular disease, and chronic obstructive pulmonary disease [6]. In fact, growing evidence suggests that short-term exposure to air pollution increases the incidence and mortality of ICH [7–11].

Tianjin, located in the Beijing-Tianjin-Hebei region, with a total resident population of 15.6 million, is the largest open coastal city in northern China [12]. In 2015, the mortality rate of ICH in Tianjin was 51.46/100,000, while the standardized mortality rate was 29.00/100,000 [13]. The mortality rate of ICH in Tianjin was decreasing in the population aged ≥ 45 years, but the mortality rate in those aged < 35 years, especially in men, was increasing [13].

The Beijing-Tianjin-Hebei region suffered the heaviest air pollution in China due to multiple factors, including the concentrations of heavy industries, geographical location, and meteorological conditions that are not conducive to the diffusion of pollutants. The annual average concentrations of major pollutants such as Fine particulate matter (PM_2.5_), Coarse particulate matter (PM_10_), Sulphur dioxide (SO_2_), and Nitrogen dioxide (NO_2_) in the Beijing-Tianjin-Hebei region are higher than the national average, and the air pollution is particularly severe after entering the heating period in winter [14]. In 2014, 1,710 severe pollution events occurred in 70 cities at or above the prefecture level in Beijing-Tianjin-Hebei and surrounding areas, accounting for 41% of the national total [15].

In order to control air pollution and protect people's health and economic development, the Chinese government implemented a series of intervention policies. In June 2014, the "Key Work of Joint Prevention and Control for Air Pollution in Beijing-Tianjin-Hebei and Surrounding Areas in 2014" was officially published. The policy required the Beijing-Tianjin-Hebei region and the surrounding areas to take unified actions to manage industrial emissions and traffic pollution jointly in the region [16]. Additionally, in response to the aggravation of air pollution in autumn and winter, the Ministry of Environmental Protection issued "The Action Plan for Air Pollution Comprehensive Control in Beijing-Tianjin-Hebei and Its Surrounding Areas in Autumn and Winter 2017–2018" in 2017 [16].

Several studies have found that comprehensive air pollution prevention measures have led to sustained improvements in air quality in the Beijing-Tianjin-Hebei region and surrounding areas [17–19]. The annual average concentrations of air pollutants (PM2.5, PM10, SO2, NO2, and CO) have decreased. Additionally, the days of heavy and serious air pollution each year have significantly decreased, while the number of days with good and excellent air quality has increased yearly.

Since the implementation of the air pollution control policy in 2014, the ICH mortality in Tianjin has decreased year by year. The reduction in air pollution exposure is expected to impact ICH deaths, but the specific impact of these policies on public health is not yet clear.

To the best of our knowledge, no previous research has yet evaluated the impact of air pollution intervention policies on cerebral hemorrhage deaths in China. As the implementation of the policies provided rare natural experiments, this study used an interrupted time series analysis to quantitatively assess the effects of the policies on the number of ICH deaths by comparing the changes in the number of ICH deaths in Tianjin before and after the implementation of the policies.

## Materials and methods

### Data

The death data of ICH from January 1, 2009, to December 31, 2020, were obtained from the All Cause of Death Registration System (CDRs) of Tianjin Center for Disease Control and Prevention, which covers the whole population of Tianjin. All deaths caused by ICH were identified using death certificates with death codes I61.x and I69.1 according to the *International Classification of Disease, 10th Revision* (ICD-10). In order to ensure the reliability of death data, the overall quality control of death certificates is carried out through a step-by-step quality audit, sampling review, and regular underreporting investigations.

The number of registered population in Tianjin comes from the Population Management Office of Tianjin Public Security Bureau. The sixth National Population Census data was provided by The National Statistics Bureau of China [20].

### Design

This study used interrupted time series (ITS) analysis, which is a quasi-experimental design. It is widely used to examine the effects of public health interventions because it is well suited for evaluating health-related outcomes over a defined period at the population level [21, 22]. During the study period, different cities in China had different air pollution control policies, making it difficult to find comparable parallel control groups [17]. Therefore, ITS analysis is appropriate for this study. ITS analysis differs from other intervention study designs in that it involves a before-after comparison within a single population rather than a comparison with a control group [23].

The pre-post design compares the results of indicators before and after the intervention, which may be confounded by biases such as secular trend, cyclical or seasonal effects, random fluctuations, and autocorrelation, making it difficult to draw reliable conclusions [24, 25]. While the interrupted time series design controls for pre- and post-intervention trends by constructing the time series with multiple measurements of the variables before and after the intervention and then using an appropriate model such as a regression model or an autoregressive integrated moving average (ARIMA) model [25].

Modeling using linear regression, logistic regression, or Poisson regression models is a common approach for ITS. Regression models require that the trend conforms to a linear hypothesis. If the regression model is incorrectly used without satisfying the linearity hypothesis, the estimate of policy impact will be biased [26]. When the time series has seasonality or autocorrelation or does not satisfy the linear hypothesis, the ARIMA model can solve these problems [27].

The pre-intervention trend is interrupted at the time of the intervention, and the predicted value that assumes no intervention and the trend continues is called counterfactual. ITS analysis evaluates the effects of interventions by comparing the difference between counterfactual and actual observations [22, 25].

The interventions evaluated were the beginning and end of two air pollution control policies. At the beginning of this study, we consulted official websites of the Chinese and local governments. During the study period, no other air pollution control policies were implemented in the Beijing, Tianjin, and Hebei regions. According to the reports on these websites [16, 28], we compiled the time and content of the four interventions studied in Table 1.

**Table 1:** Policy intervention period and contents studied in this research

Intervention period	Policy name	Content of policy intervention
From June 2014 to December 2017	Key Work of Joint Prevention and Control for Air Pollution in Beijing-Tianjin-Hebei and Surrounding Areas in 2014	Establish an expert committee for regional air pollution prevention and control; Strengthen regional cooperation, jointly manage key pollution sources, and simultaneously solve common problems; Study and formulate public policies; take the lead in implementing the particular emission limit of national air pollutants in the Beijing-Tianjin-Hebei region; Guarantee the air quality of APEC conferences in 2014
From October 2017 to March 2018	The Action Plan for Air Pollution Comprehensive Control in Beijing-Tianjin-Hebei and Its Surrounding Areas in Autumn and Winter 2017–2018	Multiple measures were implemented to strengthen the prevention and control of air pollution in winter with the core aim of improving regional ambient air quality and reducing heavily polluted weather. These measures were designed to reduce pollution emissions across the region. To ensure their effectiveness, several inspection teams were dispatched to Beijing, Tianjin, Hebei and surrounding areas for regular inspections

### Statistical analysis

The crude mortality rates for ICH and acute ICH were calculated by dividing the annual number of deaths by the population of Tianjin for the corresponding year. The age-sex standardized mortality rate was calculated using the sixth National Population Census data in 2010 as the standard population number.

In order to evaluate the impact of air pollution prevention measures on ICH and acute ICH deaths, this study based on the guidelines [27], using seasonal ARIMA models to fit the time series of the number of all ICH (ICD-10 codes I61.x and I69.1) deaths and the number of acute ICH deaths excluding sequelae of cerebral hemorrhage (ICD-10 code I69.1) in Tianjin. The models identified potential secular trends in mortality for different ICH classifications and predicted scenarios in which no interventions occurred and the trend would continue unchanged.

The seasonal ARIMA model is an analytical tool for modeling data with seasonal fluctuations in the time series. It consists of a combination of two simple models, ARIMA (p, d, q) for the non-seasonal component and ARIMA (P, D, Q) for the seasonal component. The general form of the model is ARIMA (p,d,q) × (P,D,Q)_S_ (p is the autoregressive order, q is the moving average order,and d is differencing order; P is the seasonal autoregressive order, Q is the moving average order, D is the seasonal differencing order, and S is the seasonal period) [29].

In this study, the *auto.arima()*in the *forecast* package for R is used to identify model parameters and obtain several alternative models automatically [30]. Then, the optimal model is determined based on the residual test results and the Akaike information criterion (AIC) and Bayesian information criterion (BIC).

In order to test the validity of the model, in addition to the test of autocorrelation and normal distribution of the residuals, the Ljung-Box test is also required. When the *p*-value is > 0.01, it indicates that the residual series is a white noise series, and the fitted model is able to extract almost all of the sample-related information from the series of observations, making the model significantly valid. When the *p*-value is < 0.01, the original data will be transformed to conform to normality using the Box-Cox transformation and re-fit the model.

The variables that explain the intervention in the model include the immediate change variable and the long-term trend change variable. The immediate change variable takes the value of 0 before the start of the intervention and 1 after that, while the long-term trend change variable takes the values of 0 to n, with 0 representing no intervention and 1 to n indicating the months after the intervention.

In this study, the counterfactual predicted ICH deaths were compared with the observed deaths using the beginning and end of the two air pollution control policies as the intervention time points. The effects of the air pollution control policies on ICH deaths was evaluated by calculating the immediate changes and long-term trend changes induced by the interventions.

All analyses in this study were performed in R (v.4.2.0).

## Results

**Fig. 1:**
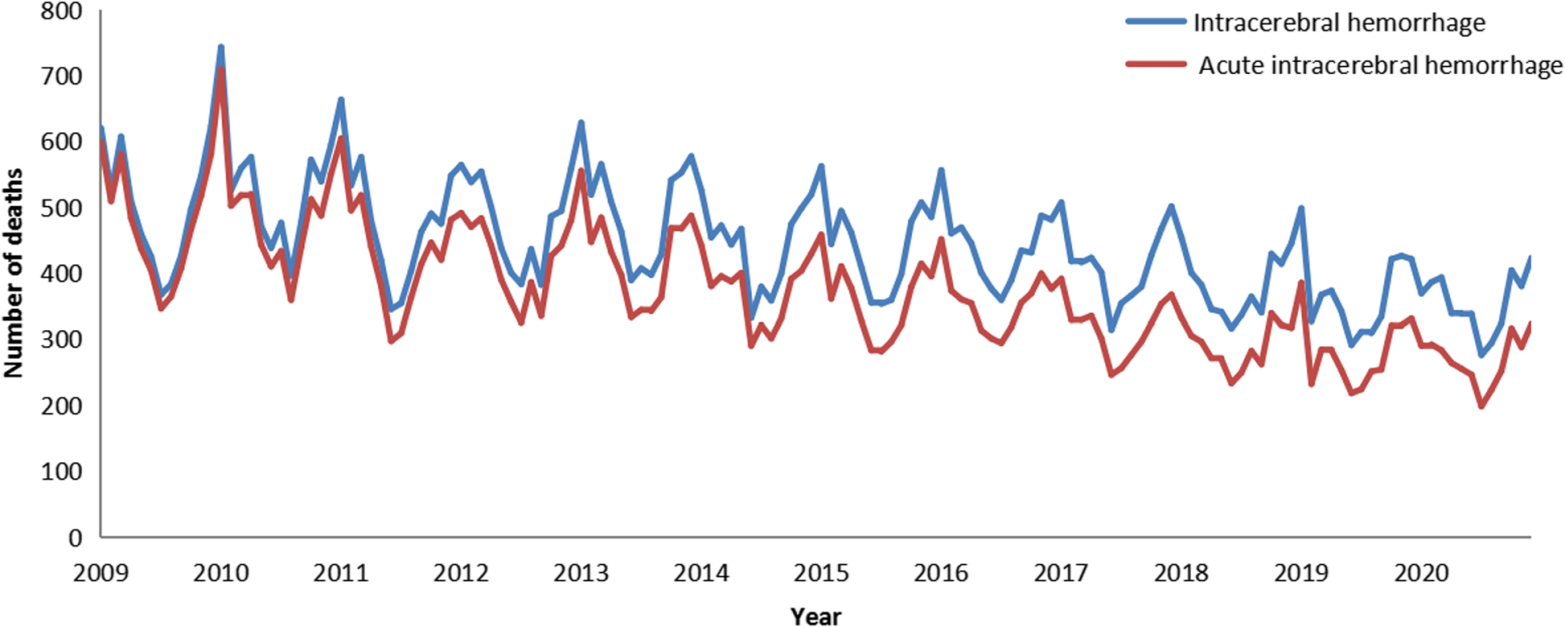
Time series of deaths from ICH and acute ICH in Tianjin from 2009 to 2020

There were 63,784 deaths due to ICH (the ICD-10 codes are I61.x and I69.1) in Tianjin from January 1, 2009, to December 31, 2020, and there were 53,788 acute ICH deaths after excluding the sequelae of cerebral hemorrhage (the ICD-10 code is I69.1). Both the crude mortality rate and age-sex standardized mortality rate of ICH showed a general downward trend. The number of deaths exhibited an annual periodicity and a seasonal trend, with peaks in winter and troughs in summer. The number of deaths, crude mortality rate, and age-sex standardized mortality rate of ICH and acute ICH are reported in Table 2. Figure 1 shows the time series of deaths from ICH and acute ICH.

**Table 2:** The number of deaths, crude mortality rate and age-sex standardized mortality rate from ICH and acute ICH in Tianjin between 2009 and 2020

Year	ICH			Acute ICH		
	Number of deaths (n)	Crude mortality Rate (1/10million)	Age-sex Standardized mortality Rate (1/10million)	Number of deaths (n)	Crude mortality Rate (1/10 million)	Age-sex standardized mortality Rate (1/10million)
2009	5986	61.43	34.21	5694	58.44	32.60
2010	6367	64.81	35.62	5883	59.89	33.06
2011	5747	58.01	31.26	5166	52.15	28.32
2012	5734	57.54	30.47	5025	50.43	26.93
2013	5974	59.50	31.25	5119	50.99	26.98
2014	5321	52.33	26.63	4470	43.97	22.57
2015	5305	51.66	25.10	4303	41.90	20.70
2016	5287	50.62	24.37	4263	40.82	20.05
2017	4973	47.36	23.03	3803	36.22	18.05
2018	4566	42.21	20.43	3476	32.14	15.91
2019	4420	39.83	18.69	3356	30.25	14.46
2020	4264	37.71	17.83	3230	28.57	13.88

### Select model

The parameters of traditional ARIMA models are determined from autocorrelation and partial autocorrelation plots, which are to some extent subjective. Therefore we identified the ARIMA model terms by using *auto.arima()* in the *forecast* package for R. The automatic algorithm was used to find the optimal model with the lowest AIC or BIC. The impact of the air pollution prevention and control policies on ICH mortality was quantitatively evaluated using the optimal ARIMA model.

The tests results indicate that the residuals are normally distributed and not significantly autocorrelated. Meanwhile, the models were subjected to the Ljung-Box test, and the residual series are white noise sequences. Our fitted models can extract almost all the sample-related information from the observation series. Therefore, the models are all significantly effective. The goodness-of-fit of models was assessed using the mean absolute percentage error (MAPE). The results are presented in Table 3.

**Table 3:** Tests of optimal models

Intervention time	Model parameter	Ljung-Box test (*P*)	AIC	BIC	MAPE (%)
ICH deaths among Tianjin residents					
June 2014	(1,0,0)(0,1,2)[12]	0.65	1320.14	1340.32	5.28
October 2017	(1,0,0)(0,1,2)[12]	0.72	1318.18	1338.36	5.24
December 2017	(1,0,0)(1,1,2)[12]	0.84	1318.87	1341.94	5.07
March 2018	(1,0,0)(2,1,0)[12]	0.97	1318.45	1338.63	5.26
ICH deaths among male Tianjin residents					
June 2014	(1,0,3)(0,1,1)[12]	0.96	1220.34	1246.29	6.29
October 2017	(1,0,0)(0,1,1)[12]	0.71	1220.64	1237.94	6.34
December 2017	(1,0,0)(0,1,1)[12]	0.67	1221.90	1239.19	6.40
March 2018	(3,0,1)(0,1,1)[12]	0.95	1220.26	1246.20	6.35
ICH deaths among female Tianjin residents					
June 2014	(0,0,0)(0,1,2)[12]	0.13	1145.14	1162.44	6.80
October 2017	(0,0,0)(0,1,2)[12]	0.15	1143.97	1161.26	6.78
December 2017	(0,0,0)(0,1,2)[12]	0.14	1142.36	1159.66	6.71
March 2018	(0,0,0)(0,1,2)[12]	0.15	1143.67	1160.97	6.69
Acute ICH deaths among Tianjin residents					
June 2014	(0,0,3)(2,1,0)[12]	0.97	1289.54	1315.48	5.52
October 2017	(0,1,1)(0,1,2)[12]	0.02	1294.96	1312.21	5.98
December 2017	(0,1,1)(0,1,2)[12]	0.02	1295.79	1296.47	6.03
March 2018	(0,1,1)(0,1,2)[12]	0.02	1296.24	1313.49	6.00
Acute ICH deaths among male Tianjin residents					
June 2014	(0,0,4)(0,1,1)[12]	0.72	1200.43	1226.38	6.78
October 2017	(0,1,1)(1,1,1)[12]	0.39	1198.95	1216.20	7.11
December 2017	(0,1,1)(0,1,1)[12]	0.45	1201.03	1215.41	7.41
March 2018	(0,0,4)(0,1,1)[12]	0.64	1198.11	1224.05	6.81
Acute ICH deaths among female Tianjin residents					
June 2014	(0,0,2)(0,1,2)[12]	0.05	1113.43	1136.49	6.99
October 2017	(0,1,1)(1,1,1)[12]	0.05	-866.86	-849.61	7.00
December 2017	(0,0,0)(1,1,1)[12]	0.27	-882.82	-865.52	6.73
March 2018	(0,0,0)(1,1,1)[12]	0.28	-880.79	-863.49	6.80

### Model prediction

Interrupted time series analysis revealed a statistically significant change in long-term trend of ICH deaths in Tianjin following the intervention in June 2014. Additionally, statistically significant immediate changes were observed following the interventions in October 2017, December 2017, and March 2018.

The number of deaths due to ICH in Tianjin decreased by 0.69 (95% CI: -1.30 to -0.07) per month under a sustained long-term trend after the intervention in June 2014. The model predicts that 54.51 (95% CI: -102.70 to -5.53) fewer ICH deaths could have been achieved with the impact of that trend during the study period. There were immediate changes that the ICH deaths in Tianjin decreased by 25.74 (95% CI: -50.62 to -0.85), 26.58 (95% CI: -52.02 to -1.14), and 30.40 (95% CI: -56.41 to -4.40) after the interventions in October 2017, December 2017, and March 2018 respectively.

There were no statistically significant differences in the immediate or long-term changes of male ICH deaths in Tianjin after the four interventions. In comparison, female ICH deaths had statistically significant long-term trend changes after the intervention in June 2014 and immediate changes after the interventions in December 2017 and March 2018.

The female ICH deaths in Tianjin decreased by 0.37 (95% CI: -0.61 to -0.13) per month under a sustained long-term trend after the intervention in June 2014. It predicts that 29.23 (95% CI: -48.19 to -10.27) fewer ICH deaths could have been achieved with the impact of that trend during the study period. There were immediate changes that the female ICH deaths in Tianjin decreased by 12.87 (95% CI: -23.7 to -1.98) and 13.08 (95% CI: -24.40 to -1.75) after the interventions in December 2017 and March 2018, respectively.

As is shown in Table 4, there was only a statistically significant immediate change in male acute ICH deaths after the intervention in October 2017, with an immediate reduction in deaths of 22.46 (95% CI: -42.76 to -2.15) in that month. While other changes in acute ICH deaths were not statistically significant according to the interrupted time series analyses.

**Table 4:** Effects of interventions on ICH and Acute ICH deaths

Intervention time	Immediate changes in ICH deaths (95%CI)	*P*	Long-term trend changes in ICH deaths (95%CI)	*P*
ICH deaths among Tianjin residents				
June 2014	-6.35 (-29.68 to 16.98)	0.59	-0.69 (-1.30 to -0.07)	0.03
October 2017	-25.74 (-50.62 to -0.85)	0.04	-0.12 (-1.10 to 0.86)	0.81
December 2017	-26.58 (-52.02 to -1.14)	0.04	-0.07 (-1.12 to 0.97)	0.89
March 2018	-30.40 (-56.41 to -4.40)	0.02	0.23 (-0.95 to 1.42)	0.70
ICH deaths among male Tianjin residents				
June 2014	-9.64 (-28.47 to 9.19)	0.32	0.33 (-0.81 to 0.16)	0.19
October 2017	-15.84 (-34.75 to 3.07)	0.10	0.10 (-0.65 to 0.84)	0.80
December 2017	-12.54 (-32.08 to 6.10)	0.21	0.07 (-0.73 to 0.88)	0.86
March 2018	-16.05 (-37.69 to 5.58)	0.15	0.25 (-0.73 to 1.24)	0.61
ICH deaths among female Tianjin residents				
June 2014	1.59 (-7.33 to 10.53)	0.73	-0.37 (-0.61 to -0.13)	0.002
October 2017	-9.35 (-19.80 to 1.09)	0.08	-0.24 (-0.65 to 0.16)	0.24
December 2017	-12.87 (-23.7 to -1.98)	0.02	-0.12 (-1.98 to 0.33)	0.59
March 2018	-13.08 (-24.40 to -1.75)	0.02	-0.06 (-0.57 to 0.45)	0.81
Acute ICH deaths among Tianjin residents				
June 2014	-5.68(-30.1 to 18.81)	0.65	-0.04 (-0.82 to 0.74)	0.92
October 2017	-23.72(-52.97 to 5.52)	0.11	0.74 (-1.49 to 2.97)	0.52
December 2017	-17.92(-49.34 to 13.50)	0.26	0.99 (-1.38 to 3.35)	0.41
March 2018	-9.82(-42.96 to 23.32)	0.56	1.29 (-1.43 to 4.01)	0.35
Acute ICH deaths among male Tianjin residents				
June 2014	-6.85 (-23.72 to 10.02)	0.43	0.06 (-0.40 to 0.52)	0.80
October 2017	-22.46 (-42.76 to -2.15)	0.03	0.59 (-0.85 to 2.04)	0.42
December 2017	-11.76 (-35.98 to 12.46)	0.34	0.83 (-1.07 to 2.74)	0.39
March 2018	-13.00 (-31.89 to 5.88)	0.18	0.78 (-0.08 to 1.64)	0.07
Acute ICH deaths among female Tianjin residents				
June 2014	-4.26 (-14.47 to 5.96)	0.41	-0.04 (-0.31 to 0.24)	0.78
October 2017	-0.004 (-0.01 to 0.004)	0.37	-0.0001 (-0.0004 to 0.0002)	0.52
December 2017	-0.006 (-0.01 to 0.001)	0.09	-0.0001 (-0.0004 to 0.0002)	0.63
March 2018	-0.005 (-0.01 to 0.001)	0.13	-0.0001 (-0.0004 to 0.0002)	0.66

**Fig. 2:**
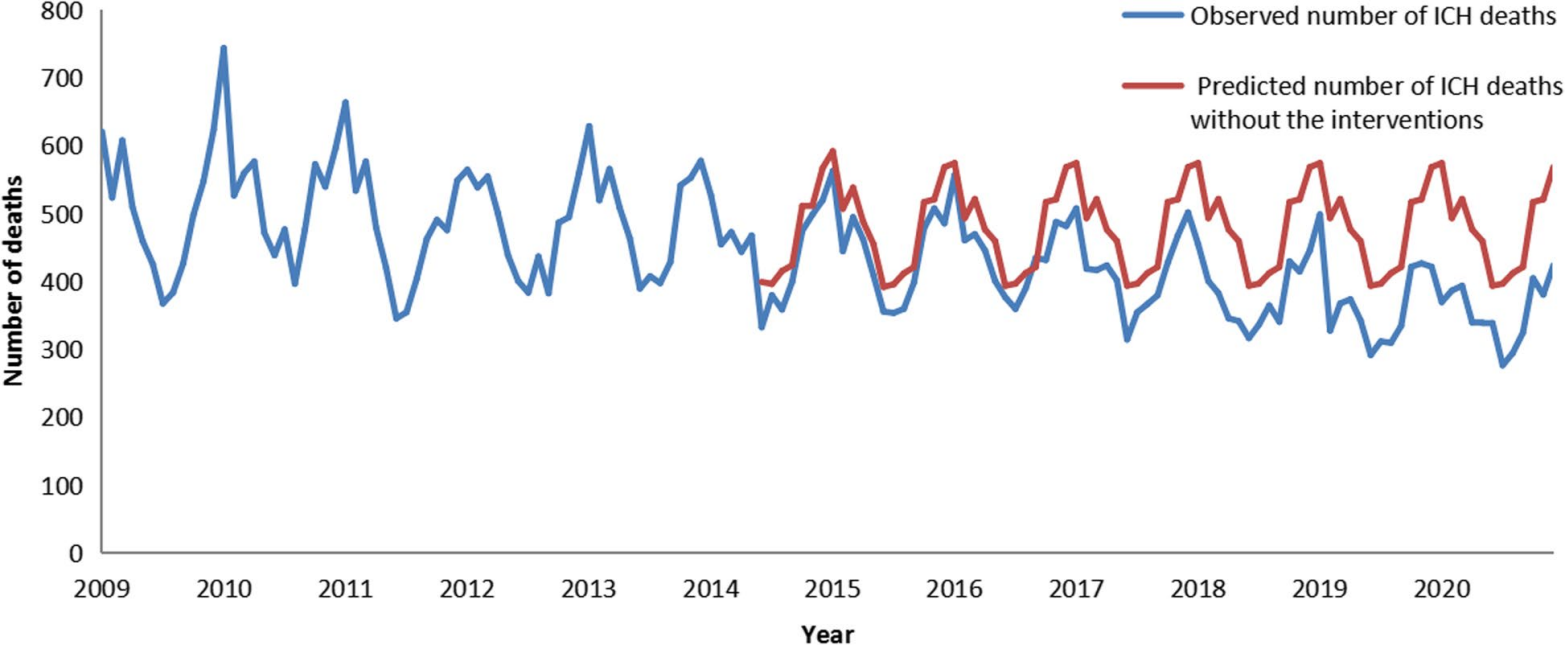
Comparison of observed and predicted ICH deaths

Without these air pollution control interventions, the number of ICH deaths between June 2014 and December 2020 is estimated to be 37,758.76. It is predicted that 5984.76 ICH deaths will be averted after the implementation of the intervention, reducing expected deaths by 15.85%. The results are presented in Fig. 2.

## Discussion

Due to the implementation of the comprehensive air pollution control policies, emissions of air pollutants in the Beijing-Tianjin-Hebei region decreased significantly [17, 31]. Several studies have shown that significant improvements in air quality have correspondingly improved human health in the study area [19, 32]. During 2009 to 2010 and 2012 to 2013, the mortality of ICH in Tianjin exhibited an upward trend. However, the mortality of ICH in Tianjin declined immediately and significantly and has continued to decline since the implementation of air pollution control policies in 2014.

The results indicated that the policy interventions had different effects on ICH mortality, possibly due to the varying goals and contents of the interventions at each stage. The long-term trend change caused by the intervention of the air pollution control policy on ICH deaths in June 2014 was statistically significant. At that time, the " Key Work of Joint Prevention and Control for Air Pollution in Beijing-Tianjin-Hebei and Surrounding Areas in 2014 " was officially published, and unified actions were carried out in Beijing-Tianjin-Hebei and surrounding regions to prevent and control air pollution [16]. Immediate changes in ICH deaths in October 2017, December 2017, and March 2018 were statistically significant. It may be associated with the government's intensified inspections and interventions to achieve air quality-related targets at the beginning and end of the policy's implementation. Therefore, the air quality in Tianjin improved at those time points, leading to significant decreases in ICH deaths.

Gender differences were observed in the effects of the interventions on ICH deaths, as shown in the stratified analysis: There was a statistically significant long-term trend change in ICH deaths for females in June 2014 and statistically significant immediate changes for females in December 2017 and March 2018. In contrast, there were no statistically significant long-term trend or immediate changes in ICH deaths for males. In addition, the results showed that none of those interventions had statistically significant effects on immediate or long-term trend changes in acute ICH mortality among all residents or female residents in Tianjin. These findings suggest that surviving ICH patients and females may be more sensitive to the protective effects of air pollution control policies.

A cohort study conducted in the UK, which accounted for a wide range of potential confounders, demonstrated that the survival rates of stroke patients would decline if they lived in areas with higher levels of air pollution [33]. Another large population-based cohort study showed that patients with hemorrhagic stroke were at a high risk for secondary cardiovascular disease. Compared with ischemic stroke patients, the cardiovascular and all-cause mortality risk in patients with hemorrhagic stroke increased significantly during follow-up [34]. Another analysis of four population-based studies concluded that ICH was associated with an approximately twofold increased risk of arterial ischemic events, ischemic stroke, and myocardial infarction [35].

Numerous studies have consistently demonstrated that women have a worse prognosis after stroke than men [36–38]. Despite higher stroke morbidity and mortality rates among men at specific ages, a greater number of women are affected by stroke due to their longer lifespan and the significantly higher incidence of stroke in the oldest age group [39, 40]. In addition, stroke-related outcomes such as disability and quality of life (QOL) are consistently worse among women than men [40].

Several related studies have shown results consistent with our study, indicating that air pollution is associated with an increased risk of ICH mortality. It was found that short-term exposure to NO_2_ was associated with increased hospital admissions for ICH in a study involving 14 major cities in China [7].There was a positive association between short-term exposure to PM_2.5_ and mortality of hemorrhagic stroke, with each 10 μg/m^3^ increase in PM_2.5_ associated with a 0.37% (95%CI: 0.07% to 0.67%) increase in mortality of hemorrhagic stroke according to a study in Beijing, China [41]. Moreover, a study in Shanghai, China, concluded similarly that the incidence of fatal ICH was associated with PM_2.5_ exposure [10]. In addition, short-term exposure to ambient SO_2_ was found to increase the risk of hospitalization for hemorrhagic stroke in a study in Guangzhou, China. The effect that SO_2_ had on the risk of hemorrhagic stroke reached the maximum value on lag 1 day, with a percentage change of 1.55% (95%CI: 0.02% to 3.11%) per 10 μg/m^3^ [42]. In the Greater Boston area of the United States, a study suggested that the risk of ICH increased after short-term exposure to ozone [43]. A study conducted in Korea found that a correlation between ozone exposure and subarachnoid hemorrhage as well as a positive association between PM_10_ and the incidence of ICH [44]. However, certain studies conducted in developed Western countries found that no significant association between air pollution and ICH deaths [45, 46]. This discrepancy may be attributed to differences in composition of air pollutants, pollution levels, meteorological factors, population susceptibility, and other factors between developed and developing countries [47].

Exposure to air pollution is an important risk factor for cardiovascular disease and leads to an increased risk of hemorrhagic stroke by biologically plausible mechanisms. Some pathophysiological alterations caused by air pollution may be related to ICH, such as arterial vasoconstriction, increased blood pressure, and increased vulnerability of brain vessel rupture due to endothelial dysfunction [48]. However, further research is required.

As shown in Fig. 1, the difference between the number of ICH and acute ICH deaths in Tianjin from 2009 to 2020 exhibited an increasing trend, indicating a decrease in acute deaths among all ICH deaths. It was probably due to residents receiving more timely treatment after the onset of ICH, which is associated with the improvement of economic development, the increase in medical resources, the promotion and education of prevention, and the adjustment of health insurance policies [49]. In recent years, Tianjin has established a number of stroke centers, which have played an essential role in the early diagnosis of stroke, reducing time to treatment and decreasing stroke mortality.

In addition to air pollution control policy, other interventions may have contributed to the reduction in ICH deaths, such as healthy lifestyle education, community health management of patients with hypertension since 2008, and the enactment of the Tianjin Act of Tobacco Control in 2012 [12, 50]. However, the implementation periods of these interventions are not consistent with the time points studied, suggesting that the reductions in ICH mortality are more likely to be related to the air pollution control policies.

In response to the outbreak of coronavirus disease 2019 (COVID-19), the Chinese government rapidly implemented a series of prevention and control measures [51]. During the epidemic, measures such as business shutdowns and vehicle restrictions reduced air pollution from industrial production and vehicle emissions [52], potentially affecting the reduction in ICH mortality. Future studies may specifically investigate the effects of COVID-19 prevention and control measures on ICH mortality in Tianjin residents.

Extensive literature has reported the association between air pollutants and stroke. However, few studies have investigated the effects of air pollution control policies on stroke and considered the types of stroke. The interventions of the Chinese government to reduce air pollution provided rare opportunities for quasi-experimental studies. The interrupted time series design is a widely used approach for evaluating policies. This design is based on observing a relatively stable population over time, which reduces the impact of inter-group differences, such as selection bias or unmeasured confounders [23]. In addition, ARIMA models account for within-group characteristics that slowly change long-term over time by modeling potential trends, such as rising economic levels and aging [23]. Another strength of this study is the use of data from The Tianjin All Causes of Death Surveillance System. The data involve a relatively fixed population and covers a long period (144 months in total). This population-based dataset provides greater statistical power to detect long-term trends and assess the health impact.

There are also some potential limitations in this study. Firstly, as an ecological study, it does not reflect the actual individual exposure levels. Therefore, individual-level confounding factors such as smoking, diet, physical activity and others cannot be entirely excluded. Secondly, there is a lack of comparison with other cities during the same period. Since corresponding air pollution control policies were implemented throughout China during the study period, it is difficult to find a comparable control group. Lastly, despite adjusting for underlying time trends, some other population-level variables not controlled for, such as obesity prevalence, prevalence of smoking, secondhand smoke exposure and influenza outbreaks, may have influenced the results, so a clear causal relationship between the reduction in ICH deaths and air pollution control measures cannot be established [23].

## Conclusions

The results demonstrated that the interventions of air pollution control policy during the study were significantly associated with reductions in ICH deaths, with greater impacts observed among females and surviving ICH patients. These findings suggest that improving air quality can reduce deaths caused by ICH. This study highlights the importance of controlling air pollution as a preventive measure for ICH mortality. Other regions with high levels of air pollution urgently need to implement similar policies for air pollution control to obtain public health benefits.

## Data Availability

The data is from the All-Cause of Death Registration System of Tianjin Center for Disease Control and Prevention. It is not publicly available but is available from the corresponding author on reasonable request.

## Abbreviations

AIC: Akaike Information Criterion
ARIMA model: Autoregressive Integrated Moving Average model
BIC: Bayesian Information Criterion
CDRs: All Cause of Death Registration System
CI: Confidence interval
DALYs: Disability adjusted life years
ICH: Intracerebral hemorrhage
ITS: Interrupted Time Series
MAPE: Mean Absolute Percentage Error
NO_2_: Nitrogen dioxide
PM_2.5_: Particulate matter with aerodynamic diameters of < 2.5 μm
PM_10_: Particulate matter with aerodynamic diameters of < 10 μm
QOL: Quality of life
SO_2_: Sulphur dioxide
YLL: Years of life lost
